# Applicability of the Broselow Pediatric Emergency Tape to Predict the Size of Endotracheal Tube and Laryngeal Mask Airway in Pediatric Patients Undergoing Surgery: A Retrospective Analysis

**DOI:** 10.7759/cureus.33327

**Published:** 2023-01-03

**Authors:** Nitinkumar Borkar, Charu Sharma, Jeswin Francis, Mayank Kumar, Subrata K Singha, Arvind Shukla

**Affiliations:** 1 Pediatric Surgery, All India Institute of Medical Sciences, Raipur, Raipur, IND; 2 Anesthesiology, All India Institute of Medical Sciences, Raipur, Raipur, IND; 3 Community and Family Medicine, All India Institute of Medical Sciences, Raipur, Raipur, IND

**Keywords:** analysis, applicability, paediatric surgery, endotracheal tube, broselow emergency tape

## Abstract

Background

This study aims to elucidate the applicability of the Broselow pediatric emergency tape in predicting the size of the endotracheal tube (ET) and laryngeal mask airway (LMA) in children of central India.

Methods

A retrospective review was conducted in the Department of Pediatric Surgery during the period of four years (January 2018 to December 2021), and all children between 1 month and 12 years of age who were admitted for routine surgery and were operated on were included. The goal was to assess the accuracy of Broselow pediatric emergency tape in predicting the size of ET and LMA in children and assess the applicability of this tape in an Indian setting based on observation and comparison with the predicted ET tube and LMA size based on the tape. The correlation was done between the predicted ET tube and LMA size and used ET tube and LMA size (the difference and mean). The Chi-square test was applied to test the difference between those matching and those not matching with their respective color zones with respect to weight, tracheal tube (LMA/ET) tube, and for both weight and tracheal tube, and then the p-value was calculated. A p-value of less than 0.05 was considered to be significant.

Results

A total of 296 patients were included in the study. There were 230 males and 66 females. A maximum number of patients were in the white zone (56 patients). A total of 112 patients (37.8%) matched the zone with their weight; 192 patients (64.8%) matched their LMA/ET tube with their respective zones; 81 patients (27.36%) matched both their weights and tracheal tube (LMA/ET) size with the predicted values as per their respective zones. Pearson’s Chi-square test was applied to assess the significance of the difference between the number of patients matching and not matching their weight, LMA/ET tube, and both weight and LMA/ET tube with their corresponding color zones as per the Broselow tape. For all the above parameters, the differences were found to be not significant for p-value <0.05.

Conclusions

Broselow tape (BT) is applicable in acute trauma settings where it can be used for estimating weight and ET/LMA sizes in an emergency situation where weight measurement is not feasible.

## Introduction

India is the second most populated country in the world, with a sixth of the world's population. As per age distribution, 28.6% of the population is less than 14 years. Pediatric patients visit the ED with various surgical and medical conditions; a few of these also require immediate intervention and resuscitation. Accurately measuring a pediatric patient's weight is crucial for effective resuscitation in pediatric emergencies, as all drug doses and equipment sizes are calculated based on the child's weight. It is impractical to measure weight during an emergency, and usually, resuscitation is done based on assumed weight or information provided by the parents. This assumption may sometimes lead to ineffective resuscitation and incorrect calculation of drug doses. In such situations, BT, designed to estimate body weight based on length, can be helpful. This tape also provides the endotracheal tube (ET) size, laryngeal mask airway (LMA) size, intercostal chest drain (ICD) size, and IV cannula size for that particular color zone to which the child belongs as per the length [1]. Among these, ET and LMA sizes must be very accurate to prevent air leaks during ventilation/resuscitation. This tape is recommended by the Advanced Trauma Life Support and the Paediatric Advanced Life Support courses, which have grown in popularity in recent years [2, 3]. Validation of the applicability of this Broselow pediatric emergency tape in predicting the size of ET and LMA has been done based on parameters of children in the United States and Europe [4-7]. As this tape has been designed using western growth references, it is likely to overestimate weight when applied to the Indian population [8-10]. This study aims to determine the applicability of the Broselow pediatric emergency tape in predicting ET and LMA sizes in children of Central India. The primary objective of this study was to assess the accuracy of the Broselow pediatric emergency tape in predicting the size of ET and LMA in children. The secondary objective was to assess the applicability of this BT in an Indian setting based on observation and comparison with the predicted ET and LMA sizes based on the tape.

## Materials and methods

This was a retrospective review conducted in the Department of Pediatric Surgery at a tertiary care institute in central India during the period of four years from January 2018 to December 2021. All children between 1 month and 12 years of age, who were admitted for routine surgery and were operated on during the study period, were included in this study. The Department of Pediatric Surgery had already conducted one study with the Institutional Ethics Committee's approval on the Accuracy of the Broselow Paediatric Emergency Tape in predicting weight in children of central India. Hence, the actual weight, height/length, and color zone category of all patients who presented to the outdoor clinics were collected and recorded. This study received separate approval from the Institutional Ethics Committee. Therefore, the case records of all these included children were reviewed, and the data on weight, height/length, color zone as per BT measurement, and predicted and used size of ET and/or LMA were recorded in an Excel sheet. Children who underwent surgery under sedation without the requirement of ET/LMA insertion, those with a height of more than 145 cm, weight of more than 36 kg, and those who were in the Red and Grey zone categories as per the tape were excluded from the study. In addition, the procedures in which air leak was present and required airway packing were excluded.

For descriptive analyses, all data were analyzed for normality. Those with a normal distribution were reported as mean with SD, and those with a skewed distribution were reported as median with range. The data were analyzed using SPSS software (version 25), and Correlation was done between the predicted ET tube and LMA sizes and actually used ET tube and LMA sizes (the difference and mean). The Chi-square test was applied to test the difference between those matching and those not matching with their respective color zones with respect to weight, tracheal tube (LMA/ET) tube, and for both weight and tracheal tube, and p-values were calculated. A p-value of less than 0.05 was considered to be significant. 

Design of BT

The Broselow pediatric tape was originally invented by emergency physicians James Broselow and Robert Luten [1]. This tape provides pre-calculated medication dosages eliminating the potential errors associated with pediatric emergent dosing preparation and administration. BT is divided into zones for medication doses and eight zones for equipment selection, as depicted in Table 1.

**Table 1:** Division of the Broselow tape into zones for medication doses and eight zones for equipment selection. ET: Endotracheal tube; LMA: Laryngeal mask airway.

Color	Estimated weight (In kilograms)	Predicted ET size	Predicted LMA size
Grey	3-5 kg	Not mentioned	
Pink	6-7 kg	3.5	1-1.5
Red	8-9 kg		
Purple	10-11 kg	4	2
Yellow	12-14 kg	4.5	2
White	15-18 kg	5	2
Blue	19-23 kg	5.5	2-2.5
Orange	24-29 kg	6	2.5
Green	30-36 kg	6.5	3

Method of using the BT 

To use the BT, the child would be in a lying-down position. Two people are needed to take the measurement. One hand is used to hold the red end of the tape so it is even with the child's head. While maintaining one hand on the red portion at the top of the child's head, the other hand is used to run the tape down the length of the child's body until it is even with his/her heels. The tape that is level with the child's heels will provide his/her approximate height in centimeters. The child's weight is taken on the weighing machine, which provides the weight in kilograms. For the recorded height of the child, the BT would give the color zone from which the predicted weight and size of LMA and ET could be predicted. The true size of the used ET or LMA tube was obtained from the intra-operative notes entered by the anesthetist in the case record. The duration between the two measurements was never more than a week. Surgeries were routine procedures of the thorax and abdomen requiring general anesthesia.

## Results

**Figure 1:**
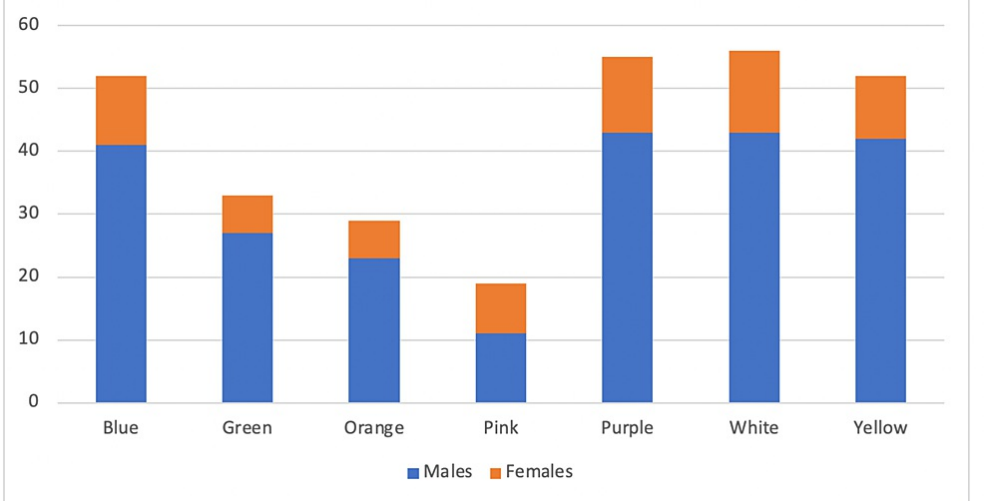
Distribution of males and females as per the color zones.

A total of 296 patients were included in the study. There were 230 males and 66 females. The maximum number of patients were in the White zone (18.9%, n=56; 43 males and 13 females), followed by the Purple zone (18.5%, n=55; 43 males and 12 females), Blue zone (17.56%, n=52; 41 males and 11 females), Yellow zone (17.56%, n=52; 42 males and 10 females), Green zone (11.1%, n=33; 27 males and six females), Orange zone (9.79%, n=29; 23 males and nine females), and Pink zone (6.4%, n=19; 11 males and eight females) (Figure 1).

**Figure 2:**
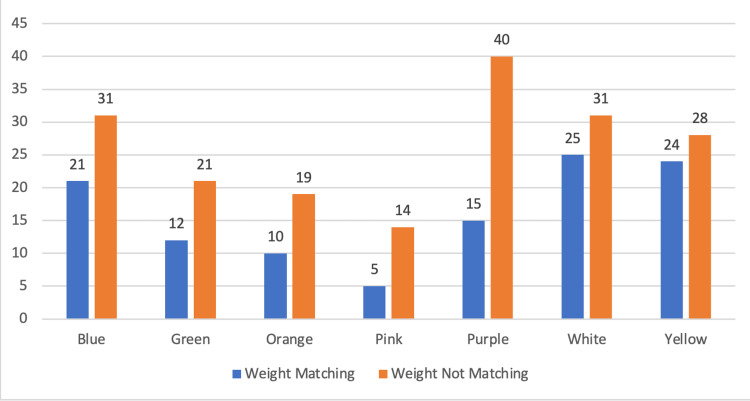
Chart showing the number of patients matching and not matching their weight with their corresponding color zones.

A total of 112 patients (37.8%) matched the zone with their weight; the maximum matching percent was in the Yellow zone, 24 patients out of 52 patients (46.15%), and the minimum matching was in the Pink zone (26.31%) (Figure 2).

**Figure 3:**
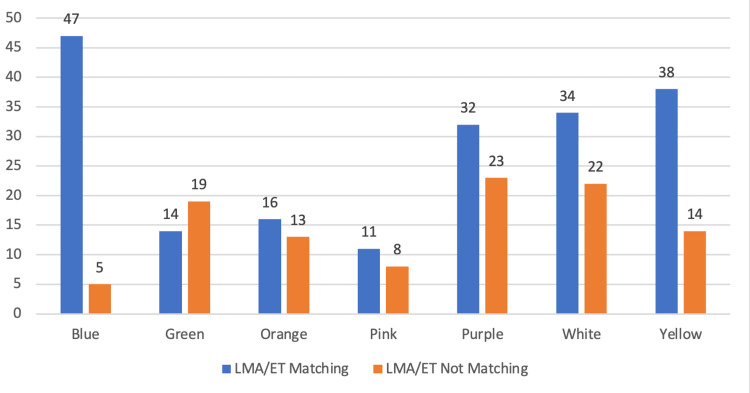
Chart showing the number of patients matching and not matching their LMA/ET with their corresponding color zones. ET: Endotracheal tube; LMA: Laryngeal mask airway.

A total of 192 patients (64.8%) matched their LMA/ET with their respective zones; the maximum matching percent was observed in the Blue zone, in which 47 among 52 patients (90.28%) matched their LMA/ET with the predicted tube size as per the weight. Conversely, the minimum percent of matching was in the Green zone (42.42%) (Figure 3).

**Figure 4:**
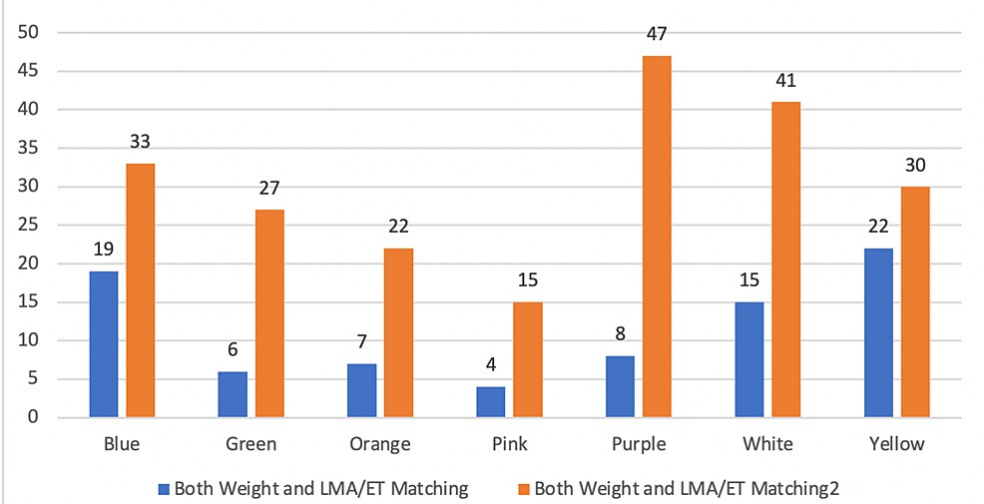
Chart showing the number of patients matching and not matching both their weight and LMA/ET with their corresponding color zones. ET: Endotracheal tube; LMA: Laryngeal mask airway.

A total of 81 patients (27.36%) matched both their weights and tracheal tube (LMA/ET) sizes with the predicted values as per their respective zones, which was maximum in the Yellow zone (42.30%) and minimum in the Purple zone (14.54%) (Figure 4).

Pearson's Chi-square test was applied to assess the significance of the difference between the number of patients matching and not matching their weight (Table 2), LMA/ET (Table 3), and both weight and LMA/ET tube (Table 4) with their corresponding color zones as per the BT. For all the above parameters, the differences were found to be not significant for p-value <0.05.

**Table 2:** Table showing Chi-square and p-values when the number of patients matching and not matching their weights with their respective color zones as per the Broselow tape.

Colour Zones	Chi-square Values	P-value	Not Significant for P<0.05
All Cases	0.036	0.849	
Blue	0.985	0.320	
Green	0.506	0.476	
Orange	0.244	0.621	
Pink	0.243	0.622	
Purple	0.655	0.418	
White	0.922	0.336	
Yellow	0.005	0.943	

**Table 3:** Table showing Chi-square and p-values when the number of patients matching and not matching their LMA/ET with their respective color zones as per the Broselow tape. ET: Endotracheal tube; LMA: Laryngeal mask airway.

Colour Zones	Chi-square Values	P-value	Not Significant for P<0.05
All Cases	0.003	0.956	
Blue	0.404	0.525	
Green	0.033	0.855	
Orange	0.658	0.417	
Pink	0.391	0.531	
Purple	0.873	0.350	
White	0.644	0.422	
Yellow	0.006	0.938	

**Table 4:** Table showing Chi-square and p-values when the number of patients matching and not matching both their weights and LMA/ET with their respective color zones as per the Broselow tape. ET: Endotracheal tube; LMA: Laryngeal mask airway.

Colour Zones	Chi-square Values	P-value	Not Significant for P<0.05
All Cases	0.906	0.316	
Blue	0.598	0.439	
Green	0.198	0.656	
Orange	0.134	0.714	
Pink	0.260	0.610	
Purple	0.346	0.556	
White	0.920	0.337	
Yellow	0.383	0.536	

## Discussion

The Broselow pediatric emergency tape, also known as the Broselow tape (BT), is a color-coded tape that is designed to estimate a child's weight, ET size, LMA size, and emergency drugs based on length [11]. It is designed for children up to approximately 12 years old with a maximum weight of roughly 36 kg. The primary use of this tape is in emergencies where the child is sick enough to record the child's weight. The correct weight estimation is necessary for managing pediatric patients in an emergency to avoid underdosing or overdosing on medications and to precisely guide the choice of ET or LMA if required. 

This tape has been designed using western growth references and could possibly overestimate the weights of Indian children. Validation of this tape has been done in various studies for the respective population worldwide [1, 4-10]. Many of these studies have validated the BT for length-weight correlation in their respective population; some of them are from India, too [1, 4-10]. On the other hand, few studies have validated the use of BT to predict the ET size [1, 8-10]. However, there are no studies on children from central India.

In a study by Hofer CK et al. in 2002 on European children, they concluded that BT accurately estimates body weight based on measured body length. However, it slightly underestimated the body weight of the whole study population. The underestimation was negligible in smaller children with a body weight of less than 20 kg. Under-estimation of body weight was pronounced in children with a body weight >20 kg [1]. They also concluded a 10% error in predicting the tracheal tube size from weight and found it to be adequate in 55% and underestimated in 39% of children [1]. They also stated that the tracheal tube size selection by the BT met clinical needs better than estimation using the standard age-based formula [1]. Jang HY et al., in 2007, on Korean children, found better agreement of ET with BT (86.9%) rather than an age-based formula [12]. They also found a good correlation of ET based on BT in shorter-height children [12]. 

In our study, the patients were divided into the eight color zones as per the BT, and predicted weight and predicted ET/LMA were then matched with true weight and true ET/LMA size for children falling in each color zone. We concluded good (37.8%) matching of weight with the color zone, and maximum matching was found in the Yellow (12-14 kg group) and Blue zones (19-23 kg). In addition, our study found good matching (64.8%) of the tracheal tube size with the color zones, with maximum matching seen in the blue zone (19-23 kg). This corroborates with the findings of Hofer CK et al., who stated that BT has 55%-77% accuracy in predicting the ET size. 

In a recent study at Bangalore, Subramanian S et al. found good relation between the length of a child with the ET size in Indian children [10]. However, this study included children aged one month to 6.5 years and those weighing 3 kg to 25 kg [10]. Asskaryar F and Shankar R added an 8% correction factor to the existing BT and used it as a weight estimation tool [13]. Mishra DG et al. concluded the BT to be reliable for weight less than 10 kg and 10-18 kg groups of urban children [14]. On evaluating the ease of using the BT in emergency situations, Agrawal S et al. concluded that BT is more convenient to use by nursing staff [15]. Our study also concluded similar results with the maximum matching of the true ET with predicted ET observed in 19-23 kg children and minimum in higher weight group (Green: 30-36 kg). In a study from Nepal, Pukar KC et al. concluded BT to be more accurate in estimating weight, ET size, and dose of adrenaline in less than 18 kg weight group, and the accuracy decreases for higher weight children [16]. Lubitz DS et al., in their study on 937 children, concluded that weight estimates generated by the tape were found to be within 15% error for 79% of the children [4]. Their study found the BT to be extremely accurate for children weighing between 3.5-10 kg and 10-25 kg [4]. Accuracy was significantly decreased for measured children who weighed more than 25 kg [4]. In a separate group of children (n = 53), the tape was shown to be more accurate than weight estimates made by residents and pediatric nurses (p less than 0.0001) [4]. Their study thus concluded that using BT is a simple, accurate method of estimating pediatric weights and drug doses and eliminates the need for memorization and calculation [4]. We also found good matching of weight in the yellow zone (12-14 kg) followed by the White zone (15-18 kg).

In a study from Indonesia, Kapuangan C et al. found BT has more predictability for ET size than the Cole formula [17]. Luten RC et al. studied 205 children undergoing ET intubation for elective surgery [5]. Their result inferred that the BT helped to select the appropriate ET size by leak pressure criterion in 77% of the cases and was within +/- 0.5 mm of the correct size 99% of the time [5]. This was significantly better (p less than 0.005) than two widely used age-based rules, which gave the correct initial size in only 47% and 9% of these cases and were within +/- 0.5 mm for 86% and 59% [5]. Thus, length-based ET selection has been found to be clearly superior to age-based rules, which are difficult to remember and require an accurate estimation of a patient's age [5]. In a prospective cross-sectional study on 548 children in Chennai by Ramarajan N et al., it was concluded that the BT overestimates weight by more than 10% in Indian children >10 kg [9]. They suggested applying a 10% weight correction factor [9]. A study by Davis D et al. found that the BT allows reliable ET size identification and should be readily available, especially in emergency situations where weight cannot be recorded [6]. Sinha M et al. concluded that length-based estimated weight using BT underestimated weight by 2.6 kg; the mean error was greatest in the highest weight category [7]. This is the same for our study also. We accept that our study has limitations. For example, it is a single-center retrospective study, and children from the urban and rural populations were not differentiated. Also, it was conducted on routine patients in outdoor clinics. However, this can serve as a pilot study for future prospective multicentric studies in the same field.

## Conclusions

Our study concludes that the BT is applicable in acute trauma settings where it can be used for estimating weight and ET/LMA sizes in an emergency situation where weight measurement is not feasible. BT also helps reduce errors due to remembering multiple complicated formulas for weight, height, and ET and LMA estimation.
